# Effect of cognitive behavioral therapy on pain, knee function, and psychological status in patients after primary total knee arthroplasty: a systematic review and meta-analysis

**DOI:** 10.1186/s12891-024-07413-1

**Published:** 2024-04-11

**Authors:** Kun Liu, Yuandong Liu, Xukai Ma, Donglin Fu, Zongqing Fan

**Affiliations:** 1 Fuyang People’s Hospital, Anhui Medical University, Fuyang, Anhui 236000 China; 2 Weifang Medical University, Weifang, Shandong 261053 China

**Keywords:** Cognitive behavioral therapy, Pain, Kinesiophobia, Catastrophizing, Function, Total knee arthroplasty, Meta-analysis

## Abstract

**Objective:**

The clinical efficacy of cognitive behavioral therapy (CBT) after Total knee arthroplasty (TKA) is still controversial, and the purpose of this meta-analysis was to evaluate the effect of CBT on pain, knee function, and psychological status of patients after TKA.

**Methods:**

We systematically searched electronic databases such as CNKI, CBM, VIP, PubMed, Cochrane Library, and EMBASE for randomized controlled studies up to February 30, 2023. Screening against inclusion criteria to select valid studies and extract data. The quality of included studies was evaluated by the Cochrane Collaboration risk-of-bias 2 (RoB 2) tool for randomized trials. Statistical analysis of the data from this study was carried out using Stata 15.1 software.

**Results:**

Finally, our meta-analysis incorporated seven randomized controlled studies of high quality, including 608 patients. The findings of the meta-analysis demonstrated a noteworthy decrease in kinesiophobia levels during the early postoperative phase in the CBT group as compared to the usual care group (WMD = -6.35, 95% CI: -7.98 to -4.72, *Z* = 7.64, *P* < 0.001). However, no statistically significant difference between the CBT and usual care groups in terms of postoperative pain as well as knee function.

**Conclusion:**

CBT may effectively reduce the level of kinesiophobia in the short term after TKA, but did not significantly relieve knee pain or improve knee function.

**Supplementary Information:**

The online version contains supplementary material available at 10.1186/s12891-024-07413-1.

## Introduction

Total knee arthroplasty (TKA) is an effective method for treating end-stage knee osteoarthritis, which can effectively alleviate pain, improve knee joint function, and enhance the quality of life of patients [1]. Despite ongoing improvements in surgical techniques, knee prosthesis designs, and postoperative rehabilitation concepts, patient dissatisfaction rates with TKA remain high, at around 20% [2, 3]. Postoperative pain and poor function are the most significant factors contributing to patient dissatisfaction[4]. Research has indicated that pain and knee function after TKA are not only linked to biological factors, but are also influenced by psychological and social factors, such as pain catastrophizing, fear of movement, patient attitudes, and pathological behavior [5, 6].

Cognitive behavioral therapy (CBT) is a purposeful, planned, and structured psychological treatment strategy that aims to gradually improve patients' maladaptive cognition and illness behaviors by correcting their negative thinking [7]. CBT can be helpful for managing pain and facilitating functional recovery after TKA [8]. A systematic review conducted by Williams et al. [9] included 75 studies involving 9,401 patients, which demonstrated the efficacy of CBT in the treatment of chronic pain in adults. Furthermore, some recent studies have demonstrated that CBT-based therapies can successfully reduce postoperative pain and pain catastrophizing levels, as well as enhance knee joint function following TKA [10, 11]. However, there are also studies indicating that CBT interventions may not improve postoperative pain and knee joint function compared to usual care [12]. Therefore, there is still controversy regarding the clinical efficacy of CBT for post-TKA.

To date, only one meta-analysis published in October 2022 has investigated the impact of CBT on postoperative pain and function after TKA [13]. It was noted that one of the included studies of the meta-analysis did not explicitly state the utilization of CBT or interventions based on CBT principles. There were also language limitations among the included studies. Additionally, since 2022, several randomized controlled trials exploring the impact of CBT on post-TKA efficacy have been published. Therefore, our study collected all randomized controlled trials (7 RCTs) [11, 12, 14–18] analyzing the impact of cognitive-behavioral therapy on post-TKA efficacy untill February 2023 and used meta-analysis to systematically analyze the effects of CBT on pain, knee joint function, and psychological status of TKA patients postoperatively, providing a more objective and reliable evaluation for clinical treatment and rehabilitation, serving as an evidence-based reference.

## Methods

This meta-analysis adhered to the guidelines set forth by the Preferred Reporting Items for Systematic Reviews and Meta-Analyses (PRISMA) in terms of its conduction and reporting [19].

The full PRISMA checklist can be found in Appendix 1. This meta-analysis has been registered in the INPLASY (registration number: INPLASY202380115).

### Literature search strategies

We employed the search strategy recommended by the Cochrane Back Review Group [20] and systematically searched electronic databases including China National Knowledge Infrastructure (CNKI), Web of Science (VIP), China Biomedical Literature (CBM), PubMed, EMBASE, and Cochrane Library. We screened and collected relevant literature before February 2023, with no language restrictions during the search process. The search strategy used a combination of MeSH terms and free terms based on the following keywords: "Cognitive Behavioral Therapy", "Behavioral Therapies", "Arthroplasty, Replacement, Knee", "Knee Replacement Arthroplasty", "randomized controlled trial", and "randomized". The search strategy can be adapted to the different databases. The English databases search strategy can be found in Appendix 2. To avoid potential omissions of relevant studies, the reference lists of included primary studies and relevant systematic reviews were also manually searched. The literature search was conducted independently by two assessors (KL and ZQF).

### Inclusion and exclusion criteria

In order to ensure the reliability of this study, rigorous inclusion and exclusion criteria were established, and two independent assessors (KL and ZQF) evaluated the literature. In case of any discrepancies, a consensus was achieved through consultation with a third assessor (DLF).

Inclusion Criteria: (1) Participants: Adult patients received primary unilateral TKA for knee osteoarthritis; (2) Interventions: Studies that explicitly state that they are based on cognitive behavioral therapy principles or use cognitive behavioral therapy methods; (3) Comparisons: No treatment or other interventions alone(non-CBT intervention); (4) Outcomes: Includes one of the following outcome indicators: Visual Analog Scale (VAS), numerical rating scale (NRS), American knee society knee score (KSS), Hospital for Special Surgery Knee Rating Scale (HSS), Tampa Scale of Kinesiophobia (TSK), Pain Catastrophizing Scale (PCS); (5) Study design: The literature type must be a RCT. Exclusion Criteria: (1) Animal experiments, case reports, conference abstracts, clinical trial registrations, reviews, and meta-analyses; (3) Not including any of the above outcome indicators; (4) Significantly incomplete outcome data.

### Data extraction

Two separate researchers (KL and ZQF) collected the following information from the included studies using a pre-designed standardized form: (1) Essential details of the study: (publication year, primary author, country, study type); (2) Demographic information of the participants: (age, gender, sample size); (3) Description of CBT intervention: (including duration, frequency, the background of implementers, and method of implementation); (3) Primary outcome measures: Pain intensity (VAS score, NRS score); Secondary outcome measures: Knee joint function (KSS score, HSS score), Psychological status (TSK score, PCS score). When data could not be directly obtained, we contacted the authors for requests or used Engauge Digitizer software for extraction [21]. If the statistical description of the data does not meet our requirements, we will convert the form of data description [22]. If outcome measures are reported with varying results due to differing follow-up times, the data will be grouped into subcategories with similar follow-up periods. Evaluations conducted within 3 months after surgery will be labeled as short-term follow-up, while evaluations undertaken at 1 year after surgery will be identified as long-term follow-up. Any discrepancies in data extraction will be discussed and resolved by two assessors (KL and ZQF) or through consultation with a third researcher (DLF).

### Quality assessment of the studies

Since all the included studies were randomized controlled trials, the Cochrane Collaboration risk-of-bias 2 (RoB2) tool was used to evaluate the methodological quality of each study. The assessors made high, unclear, or low-risk evaluations for each item based on the assessment criteria.. Since the authors of this study were familiar with the content of the included literature, blinding of the authors, institutions, and journals of the included studies was not feasible during the risk of bias assessment. The assessment was conducted independently by two assessors (KL and ZQF), and any disagreements were resolved through discussion with a third assessor (DLF).

### Statistical analysis

The summary effect sizes for continuous variables were expressed as weighted mean differences (WMD) or standardized mean differences (SMD) (when there were inconsistencies in measurement units or methods), along with a 95% confidence interval. For binary variables, the summary effect sizes were expressed as relative risks (RR) and a 95% confidence interval. Hypothesis testing was performed using the Z-test, and the statistical heterogeneity among the included studies was analyzed using the Cochrane *Q* test and *I*^*2*^ statistics (Der Simonian Laird). If there was no significant heterogeneity among the studies (*I*^*2*^<50% or *P*>0.1), a fixed-effect model was used for meta-analysis. If there was significant heterogeneity (50%≥*I*^*2*^ or *P*≤0.1), a random-effects model was used to pool the effect sizes. Due to the limited number of included studies, we did not use meta-regression to assess potential confounding factors that may affect the combined effect size. To evaluate the stability of the results, sensitivity analyses were undertaken by omitting one study at a time and noting changes in the combined effect size of the primary outcome measure. We did not draw funnel plots due to the small number of included studies (<10) but used the Begg's test and Egger's test to assess potential publication bias for studies reporting primary outcome indicators [23]. All statistical analyses were conducted using Stata software (Version 15.1; StataCorp LLC, College Station, USA), with a significance level of *P*<0.05 indicating statistically significant differences.

## Results

### Literature screening

**Fig. 1:**
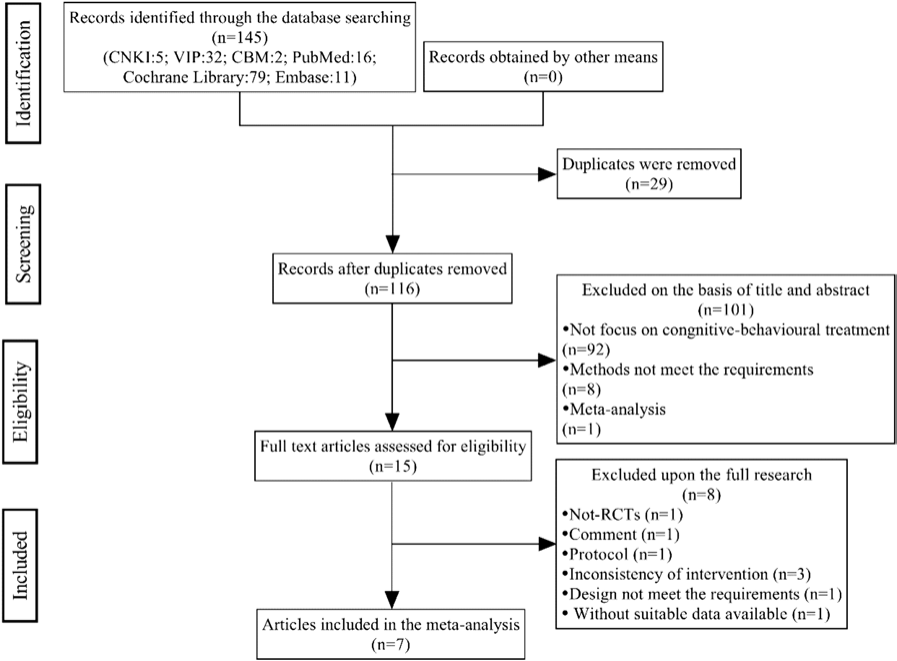
Flow diagram of the detailed literature screening process

After implementing our search strategy, we identified a total of 145 potentially relevant studies from six electronic databases. Moreover, we conducted a manual search of the reference lists of pertinent studies, yielding no further pertinent studies. Specifically, we found 5 studies from CNKI, 16 studies from PubMed, 79 studies from Cochrane Library, 11 studies from Embase, 32 studies from VIP, and 2 studies from CBM. The process of literature screening is illustrated in Fig. 1.

### Quality assessment of the studies

**Fig. 2:**
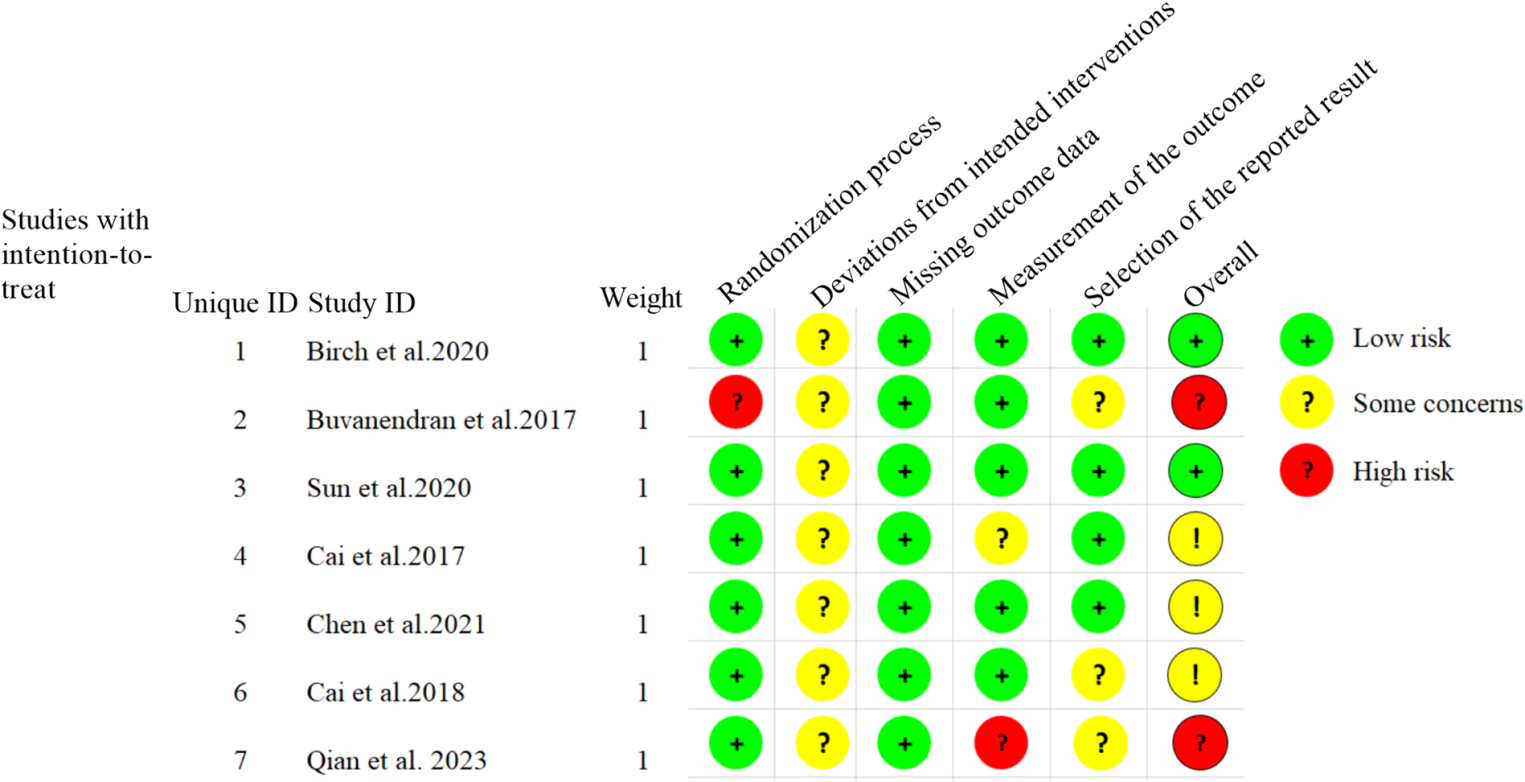
Assessment of risk of bias items for each included study

**Fig. 3:**
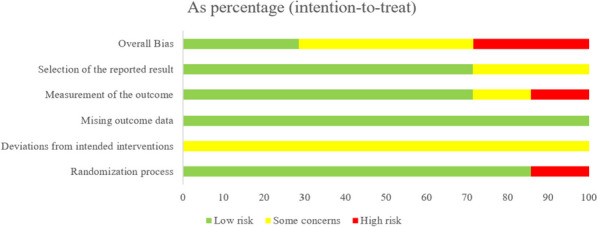
Proportions of authors' evaluations regarding the risk of bias for each item in the included studies

In this analysis, we included 7 articles, all of which were described as randomized controlled trials.The assessment of risk of bias for each study in each evaluation domain, as well as the overall comparison of the proportion of studies with low risk, some concerns, and high risk in each domain, are shown in Figs. 2 and 3.

### Basic characteristics and demographic information of the studies

This meta-analysis included 7 studies with a total of 608 patients, comprising 216 males and 392 females. One study [12] had an average patient age of ≥ 70 years, while the remaining studies had an average patient age between 55 and 70 years. The main characteristics of the included studies are presented in Table 1. Four studies [11, 14, 16, 18] explicitly stated that CBT was used as the intervention measure, three studies [12, 15, 17] indicated that the intervention measures were based on CBT principles. CBT was delivered through face-to-face and remote telemedicine sessions, with each study conducting CBT interventions more than 4 times. CBT was implemented by relevant medical professionals, such as doctors, nurses, psychotherapists, and physical therapists, either independently or in combination. Usual care was used as the control group in all studies. Detailed information of the intervention measures in the included studies as shown in Table 2. Among the extracted indicators, two studies used VAS scores, while three studies used NRS scores to evaluate the intensity of pain. Three studies used PCS to evaluate pain catastrophizing, and three studies used TSK to evaluate kinesiophobia. None of the included studies reported any adverse events related to CBT. The primary outcome measures in the included studies are presented in Table 3.

**Table 1:** Basic characteristics of the included studies

Study (Author, year)	Country	Study type	Simple size (N)	Gender (M/F, N)	Age (years, Mean ± SD)	Educational level (N)
Birch et al. (2020) [15]	Denmark	RCT	EG: 31 CG: 29	EG: 9/22 CG: 11/18	EG: 66 ± 9 CG: 66 ± 10	EG: < 3 years:18 ≥ 3 year:13 CG: < 3 years:14 ≥ 3 year:15
Buvanendran et al. (2017) [16]	US	RCT	EG: 40 CG: 37	EG: 14/26 CG: 12/25	EG: 64.7 ± 8.9 CG: 63.3 ± 13.3	NM
Sun et al. (2020) [17]	China	RCT	EG: 42 CG: 38	EG: 23/19 CG: 17/21	EG: 57.8 ± 8.7 CG: 60.2 ± 8.2	EG: < 6 years: 40 > 6 years:2 CG: < 6 years:36 > 6 years:2
Cai et al. (2017) [14]	China	RCT	EG: 54 CG: 54	EG: 23/31 CG: 20/34	EG: 62.42 ± 6.59 CG: 63.94 ± 6.58	EG: ≤ 9 years: 24 > 9 years: 30 CG: ≤ 9 years:27 > 9 years:27
Chen et al. (2021) [12]	China	RCT	EG: 42 CG: 41	EG: 11/31 CG: 13/28	≥ 70	EG: ≤ 5 years: 40 > 5 years:2 CG: ≤ 5 years: 38 > 5 years:3
Cai et al. (2018) [11]	China	RCT	EG: 50 CG: 50	EG: 18/32 CG: 20/30	EG: 65.26 ± 8.30 CG: 66.18 ± 7.04	EG: ≤ 6 years:10 > 6 years: 40 CG: ≤ 6 years:16 > 6 years:34
Qian et al. (2023) [18]	China	RCT	EG: 51 CG: 49	EG: 20/31 CG: 17/32	EG: 58.35 ± 5.40 CG: 58.31 ± 5.37	NM

*RCT* Randomized Controlled Trial, *EG* Experimental group, *CG* Control Group, *NM* Not Mentioned, *M/F* male/female

**Table 2:** The specific details of the interventions

Study (Author, year)	Exercise group	Control group
Birch et al. (2020) [15]	EG: CBT + UC CBT: Psychologists and physiotherapists conducted approximately six face-to-face sessions, each lasting around 45 min. There were two sessions before and four sessions after the surgery (1–2 days, 2 weeks, 4 weeks, and 12 weeks post-surgery)	CG: UC UC: During hospitalization, patients receive regular rehabilitation guidance and care. Nurses, physiotherapists, and the operating surgeon provide rehabilitation guidance during four follow-up visits (1 week, 2 weeks, 4 weeks, and 12 weeks postoperatively)
Buvanendran et al. (2017) [16]	EG: CBT CBT: Four CBT sessions were conducted via telehealth by researchers within one week before the operation	CG: UC UC: The patients receive usual care before the surgery, as well as regular guidance on rehabilitation exercises postoperatively
Sun et al. (2020) [17]	EG: CBT + UC Psychologists conducted a total of six face-to-face meetings, with each session lasting approximately 30 min. Three meetings took place prior to the surgery (2–3 days before, 1 week before, and 2 weeks before), and the other three meetings occurred post-surgery (2 days, 5–7 days, and 2 weeks postoperatively)	CG: UC UC: Patients receive usual care, and upon admission, nurses provide standard education. Prior to the surgery, there is communication between the surgeon, physiotherapists, and anesthesiologist with the patient regarding the surgical procedure, anesthesia, and postoperative rehabilitation
Cai et al. (2017) [14]	EG: CBT + UC CBT: During the postoperative hospitalization period, a team consisting of doctors, psychologists, rehabilitation therapists, and nurses conducts face-to-face sessions with the patient twice a day, lasting approximately 30–40 min each	CG: UC UC: During the postoperative hospitalization period, patients receive routine nursing interventions such as health education and rehabilitation guidance
Chen et al. (2021) [12]	EG: CBT + UC CBT: During the hospitalization period, doctors and nurses (trained by psychologists and physiotherapists) conducted five face-to-face sessions (on admission, 1 day before surgery, 2–3 days post-surgery, 4–5 days post-surgery, and the day before discharge)	CG: UC UC: During the hospitalization period, routine nursing care was provided following standard nursing protocols
Cai et al. (2018) [11]	EG: CBT + UC CBT: During the hospitalization period, physiotherapists and psychologists provide four face-to-face meetings, each lasting approximately 40 min (1–2 days, 2–4 days, and 3–4 days postoperatively)	CG: UC UC: During the hospitalization period, routine postoperative rehabilitation guidance and care are provided. After discharge, there will be biweekly contact with a physiotherapist for rehabilitation exercise guidance
Qian et al. (2023) [18]	EG: CBT + UC CBT: From the first day postoperatively until discharge, the intervention team consisting of doctors, nurses, and physiotherapists conducts CBT intervention meetings twice daily, with each meeting lasting 20–30 min	CG: UC UC: Patients receive routine postoperative care and functional rehabilitation guidance

*EG* Experimental group, *CG* Control Group, *CBT* Cognitive Behavioral Therapy, *UC* Usual Care

**Table 3:** Primary outcome indicators

Study (Author, year)	Primay indicators	Time point
Birch et al. (2020) [15]	VAS during activity and rest, PCS,	3 months and 12 months postoperatively
Buvanendran et al. (2017) [16]	NRS during activity and rest	3 months postoperatively
Sun et al. (2020) [17]	VAS during activity and rest, PCS, HSS	1 month, 3 months, and 12 months postoperatively
Cai et al. (2017) [14]	KSS knee function score, TSK	1 month, 3 months, and 6 months postoperatively
Chen et al. (2021) [12]	VAS during activity, HSS	2 days, 5 days, 3 months, 12 months postoperatively; day of discharge
Cai et al. (2018) [11]	TSK, NRS during rest, TSK, PCS, HSS	1 month, 6 months postoperatively
Qian et al. (2023) [18]	NRS, TSK	2 days, 7 days, and 1 month postoperatively

*NRS* Numerical Rating Scale, *VAS* Visual Analog Scale, *PCS* Pain Catastrophizing Scale, *KSS* American knee society score, *TSK* Tampa Scale of Kinesiophobia, *HSS* Hospital for Special Surgery Knee Rating Scale

### Meta-analysis results

#### Primary outcome indicators

##### The effect of CBT on pain intensity in patients after TKA

**Fig. 4:**
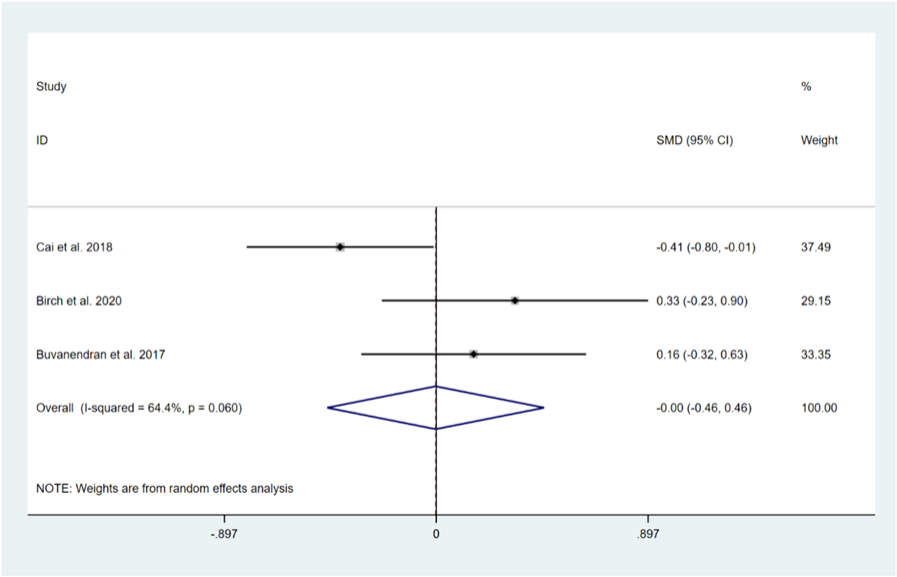
Forest plot comparing pain intensity at rest after TKA between the CBT group and the usual care group in the short-term follow-up

**Fig. 5:**
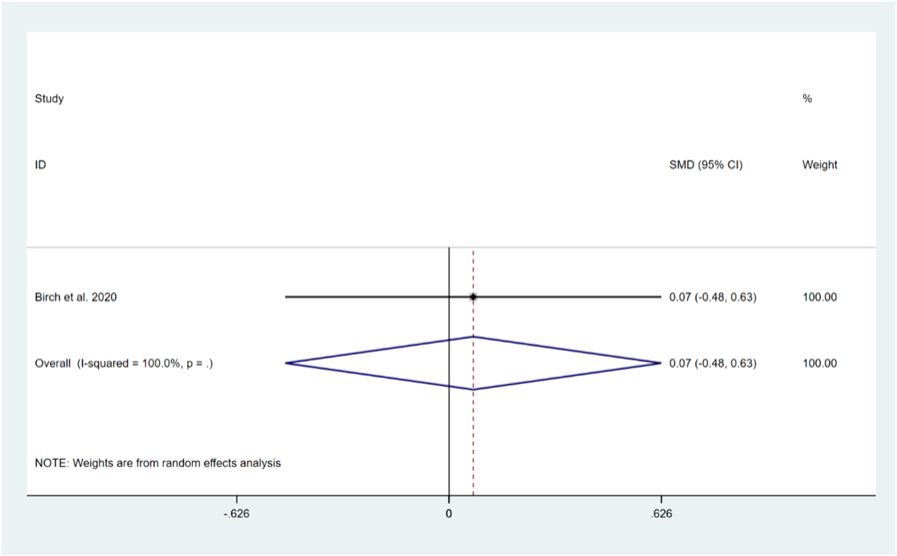
Forest plot comparing pain intensity at rest after TKA between the CBT group and the usual care group in the long-term follow-up

Six studies, involving a total of 500 patients, reported on the pain experienced by patients after TKA [11, 12, 15–18]. Among them, three studies reported on pain experienced during the short-term period (within 3 months after surgery) in the resting state [11, 15, 16]. The results of the heterogeneity test revealed significant heterogeneity among this studies (*I*^*2*^=64.4%, *P*=0.060). As a result, a random-effect model was used for meta-analysis. The combined effect size results showed that there was no significant difference between the two groups (SMD=-0.00, 95%CI: -0.46 to 0.46, Z=0.01, *P*=0.991). Only one study reported on pain experienced during the long-term follow-up (1 year after surgery) in the resting state of the knee [15], and the results of the study showed no significant difference between the two groups (SMD=0.07, 95%CI: -0.48 to 0.63, *Z*=0.25, *P*=0.801). This indicates that CBT intervention, compared with usual care, did not significantly alleviate pain intensity in the knee joint at rest after TKA, as shown in Figs. 4 and 5.

**Fig. 6:**
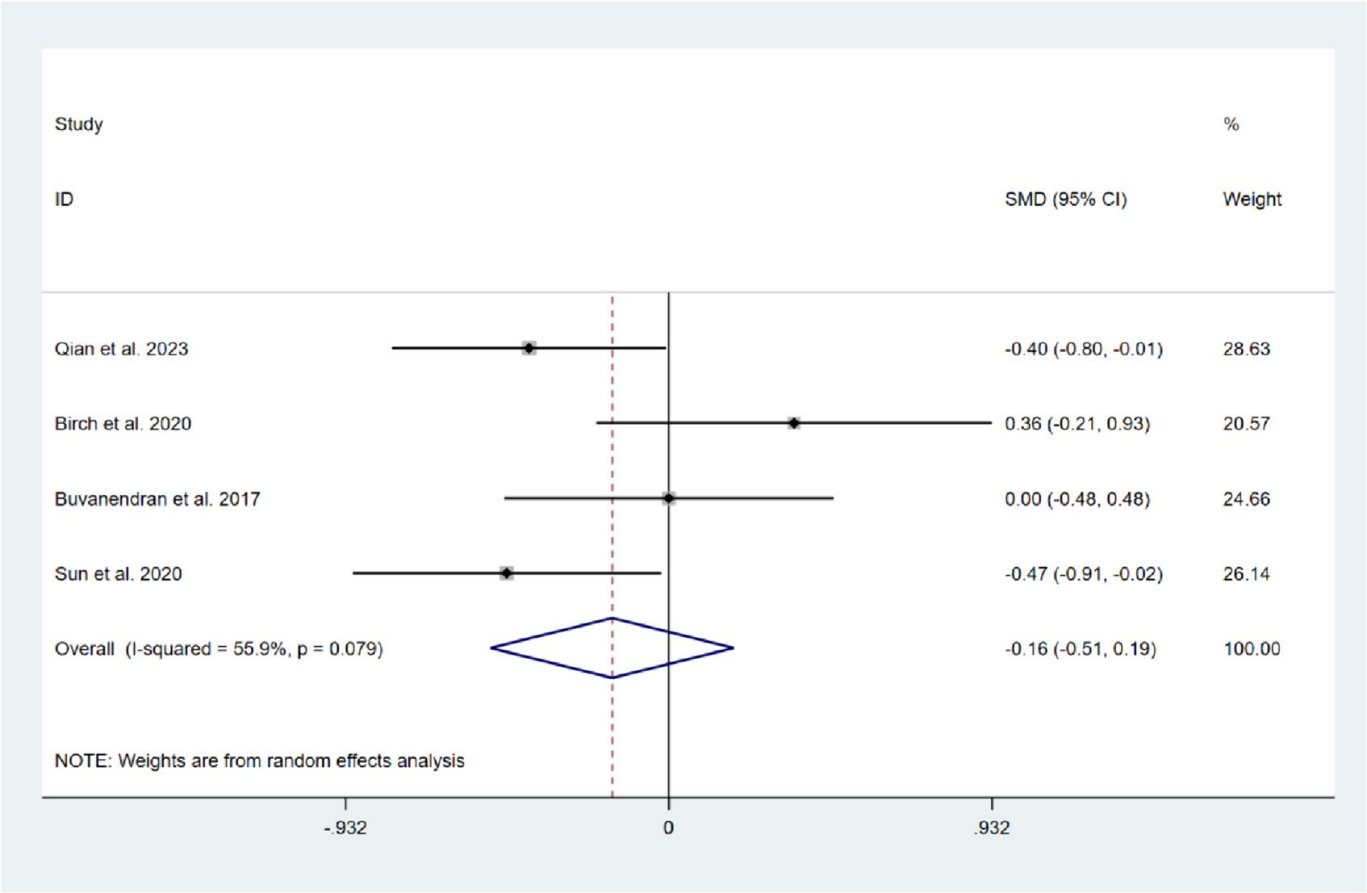
Forest plot comparing pain intensity during activity between the CBT group and the usual care group after TKA in short-term follow-up

**Fig. 7:**
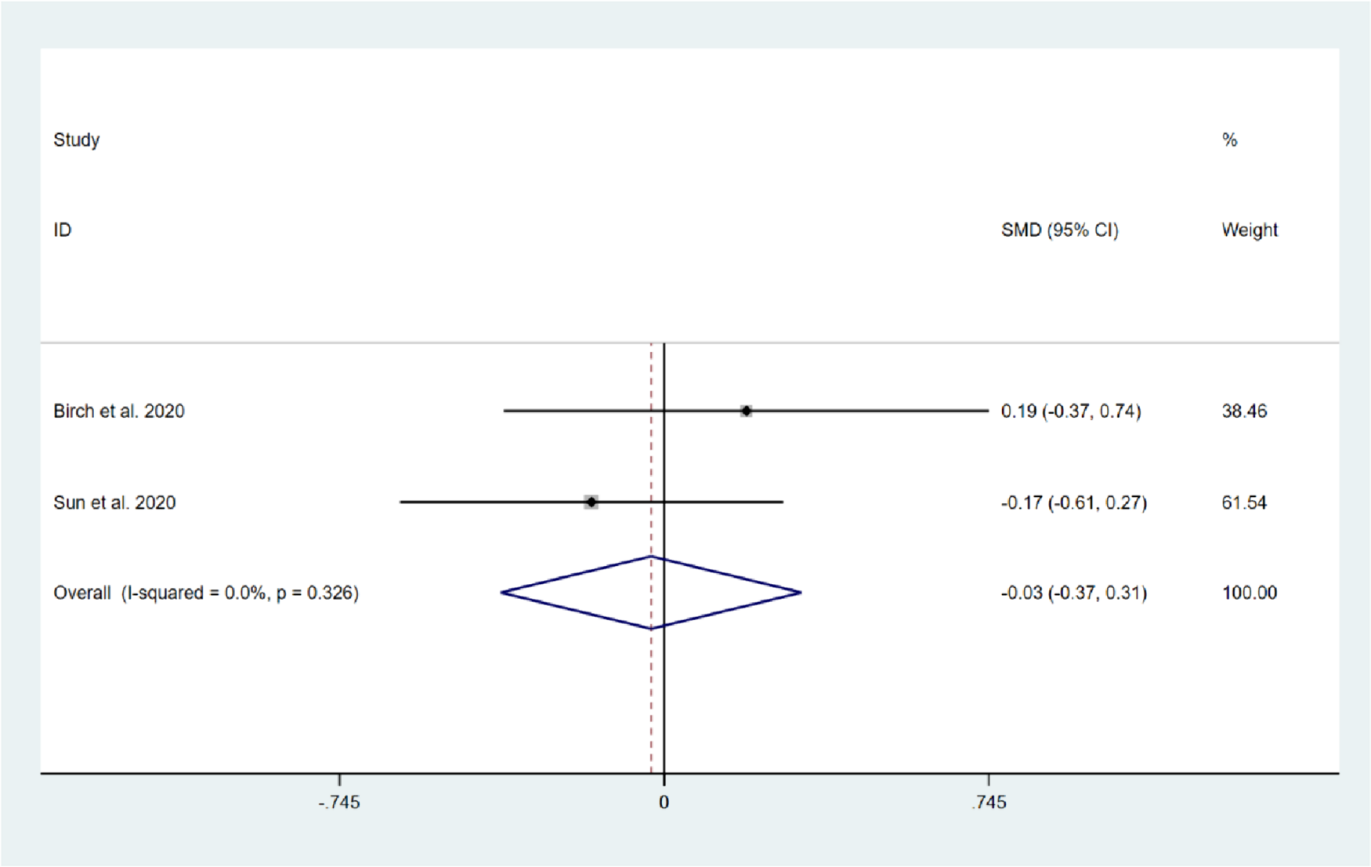
Forest plot comparing pain intensity during activity between the CBT group and the usual care group after TKA in long-term follow-up

Four studies reported on the pain experienced during activity in the short-term period [15–18], and the results of the heterogeneity test demonstrated a moderate degree of heterogeneity among these studies (*I*^*2*^ = 55.9%, *P* = 0.079). The random-effects model was selected to compute the combined effect sizes. The results showed that there was no significant difference in pain intensity during activity between the two groups (SMD = -0.16, 95% CI: -0.51 to 0.19, Z = 0.91, *P* = 0.362). Two studies reported on pain experienced during activity in the long-term follow-up [15, 17], and the results of the heterogeneity test demonstrated no significant heterogeneity between the two studies (*I*^*2*^ = 0.0%, *P* = 0.326). Fix-effect model was selected to pool the effect sizes, no significant difference was found between the two groups (SMD = -0.03, 95% CI: -0.37 to 0.31, Z = 0.17, *P* = 0.865). This indicates that compared with usual care, CBT intervention did not significantly alleviate pain intensity in the knee during activity after TKA, as shown in Figs. 6 and 7.

#### Secondary outcome measures

##### The impact of CBT on knee function in patients after TKA

**Fig. 8:**
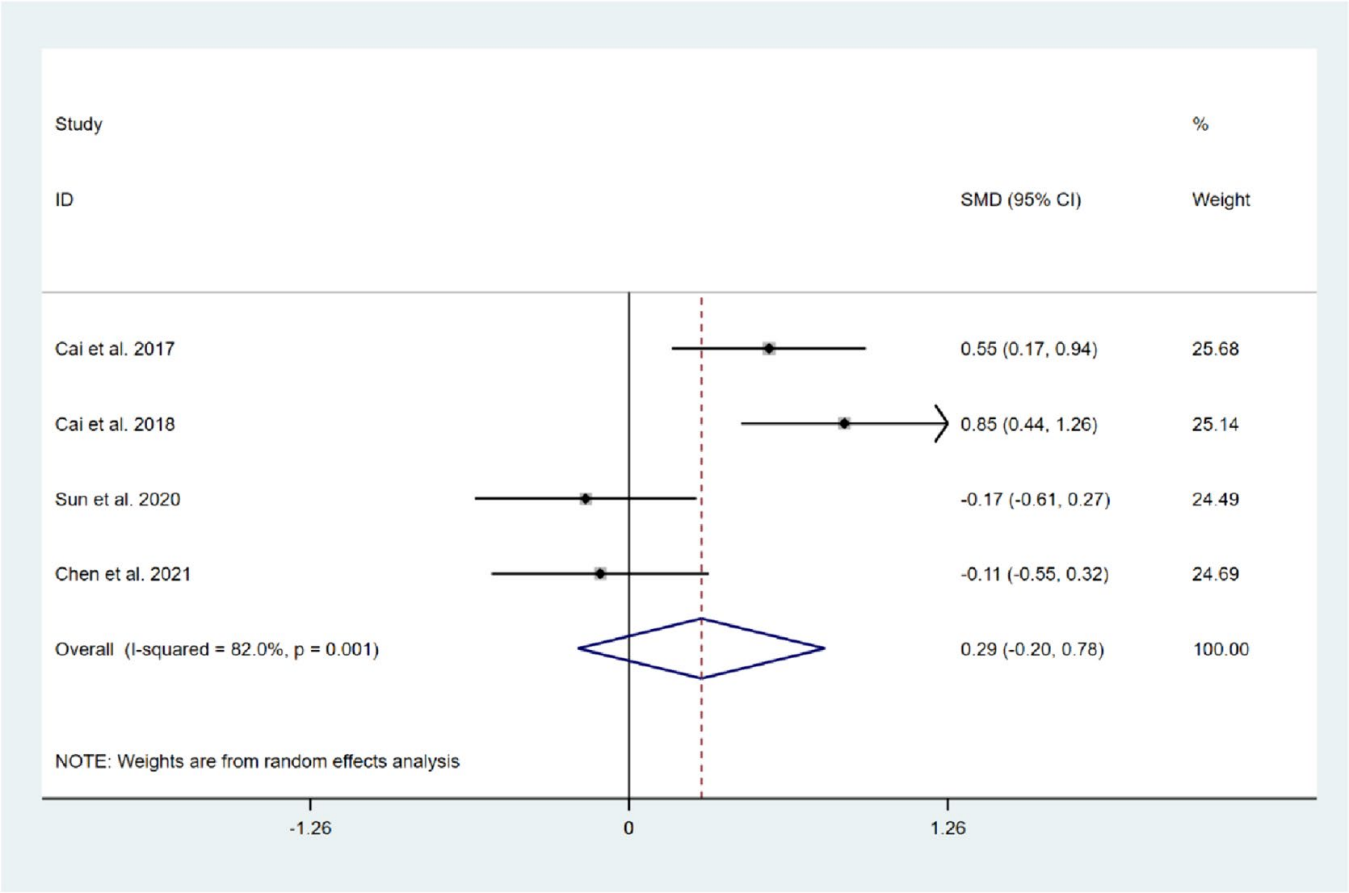
Forest plot comparing knee joint function between the CBT group and the usual care group after TKA in short-term follow-up

**Fig. 9:**
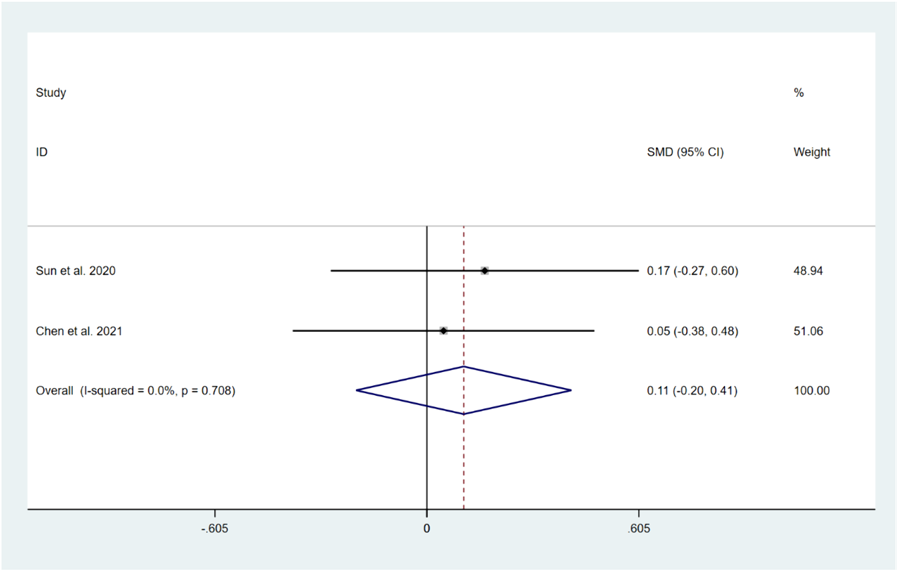
Forest plot comparing knee joint function between the CBT group and the usual care group after TKA in long-term follow-up

A total of four studies, comprising 371 patients, reported on the impact of CBT on knee joint function in patients after TKA compared with usual care [11, 12, 14, 17]. Among them, four studies reported on knee joint function recovery in the short-term period [11, 12, 14, 17], and the results of the heterogeneity test demonstrated significant heterogeneity among these studies (*I*^*2*^ = 82.0%, *P* = 0.001). Random-effects model was selected to compute the combined effect sizes, and the results showed that there was no significant difference between the two groups (SMD = 0.29, 95% CI: -0.20 to 0.78, *Z* = 1.15, *P* = 0.252). Two studies reported on knee joint function recovery during long-term follow-up [12, 17], and no significant heterogeneity was found between the two studies (*I*^*2*^ = 0.0%, *P* = 0.708). The combined results of the fixed effects model showed no significant differences between the two groups (SMD = 0.11, 95% CI: -0.20 to 0.41, *Z* = 0.67, *P* = 0.503). This indicates that CBT intervention compared with usual care was not effective in promoting knee joint function recovery after TKA, as shown in Figs. 8 and 9.

##### The impact of CBT on the psychological status of patients after TKA

**Pain Catastrophizing Scale (PCS) score**

**Fig. 10:**
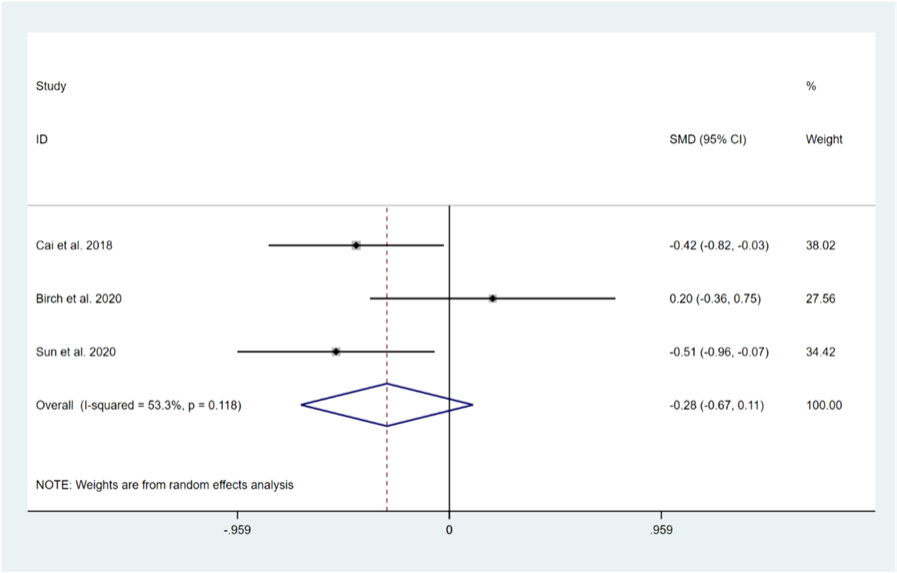
Forest plot comparing PCS scores between the CBT group and the usual care group after TKA in short-term follow-up

**Fig. 11:**
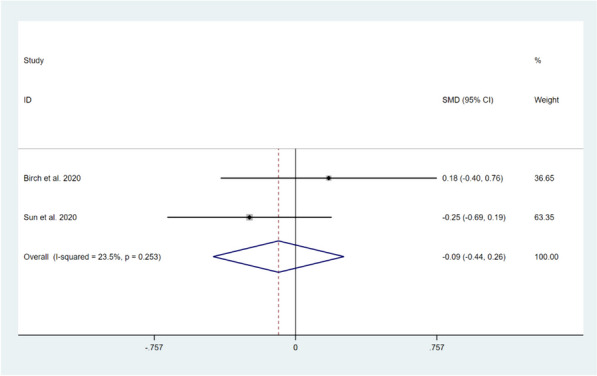
Forest plot comparing PCS scores between the CBT group and the usual care group after TKA in long-term follow-up

Three studies involving 240 patients reported on the impact of CBT on pain catastrophizing levels in patients after TKA compared with usual care [11, 15, 17]. Among them, three studies reported on the Pain Catastrophizing Scale (PCS) score in the short-term period [11, 15, 17], and the Random-effects model was selected to compute the combined effect sizes (*I*^*2*^ = 53.3%, *P* = 0.118). The results showed that there was no significant difference between the two groups (SMD = -0.28, 95% CI: -0.67 to 0.11, *Z* = 1.42, *P* = 0.155). Two studies reported on PCS score in long-term follow-up [15, 17], and no significant heterogeneity was found between the two studies (*I*^*2*^ = 23.5%, *P* = 0.253). Fixed effects model was applied to pool effect sizes, and the results showed that no significant differences between the two groups (SMD = -0.09, 95% CI: -0.44 to 0.26, *Z* = 0.51, *P* = 0.610). This indicates that CBT intervention was not effective in improving the pain catastrophizing levels of patients after TKA compared to usual care, as shown in Figs. 10 and 11.

**Tampa Scale of Kinesiophobia (TSK) score**

**Fig. 12:**
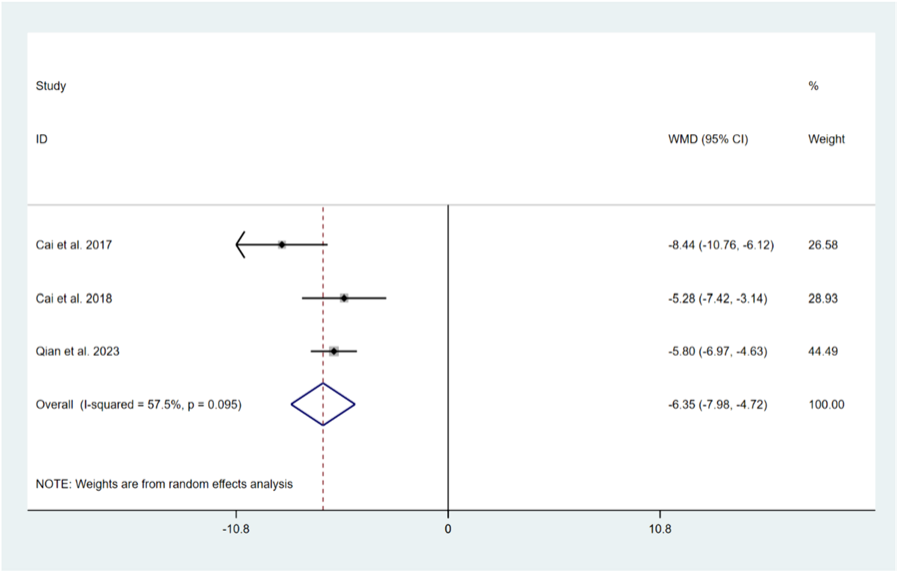
Forest plot comparing TSK scores between CBT intervention and usual care groups after TKA

Three studies involving 308 patients reported on the impact of CBT intervention on postoperative movement fear in the short-term period after TKA [11, 14, 18]. The heterogeneity test showed moderate heterogeneity among the included studies (*I*^*2*^ = 57.5%, *P* = 0.095), therefore, a random-effects model was chosen for the meta-analysis. The pooled effect size indicated that there was a statistically significant difference in TSK scores between the CBT and the usual care groups (WMD = -6.35, 95% CI: -7.98 to -4.72, *Z* = 7.64, *P* < 0.001). This indicates that CBT intervention is more effective than usual care in improving postoperative movement fear in patients after TKA, as shown in Fig. 12.

Indicators of outcome for studies included in this meta-analysis are provided in Appendix 3.

### Sensitivity analysis and publication bias

In the sensitivity analysis and publication bias assessment of the primary outcome measure, pain intensity after TKA, we took into account the limited number of studies in the long-term follow-up comparison and therefore only evaluated the short-term outcome measure. The sensitivity analysis results revealed that omitting any individual study had no significant impact on the pooled effect size, indicating the stability of the results (Pain intensity at rest and activity). Publication bias was assessed by the Begg's test and Egger's test, and the results suggested that there was no substantial publication bias across these studies (pain during resting state: Egger's test *P* = 0.194, Begg's test *P* = 0.296; pain during active state: Egger's test *P* = 0.097, Begg's test *P* = 0.308).

## Discussion

Cognitive-behavioral therapy (CBT) is a purposeful, planned, and structured psychological treatment strategy that can be implemented by various healthcare professionals. Its goal is to gradually change patients' maladaptive and erroneous thinking patterns and behaviors, ultimately leading to psychological and physical recovery [24]. However, the effectiveness of CBT in postoperative management of TKA is still uncertain [12]. Therefore, we conducted this meta-analysis to investigate whether CBT can reduce pain, improve knee joint function, and enhance psychological status in patients after TKA.

The CBT intervention after TKA is aimed not only at treating pain and functional impairments, but also at changing patients' cognition and maladaptive behaviors, which can have an impact on their postoperative recovery process [25]. Our study results showed that CBT can alleviate the level of postoperative short-term kinesiophobia in patients.

Recent studies examining the impact of CBT on pain intensity have yielded conflicting results [15, 17]. Five of the studies in this meta-analysis reported on the effect of CBT on postoperative pain intensity [11, 15–18]. The combined effect size indicated that CBT intervention did not significantly reduce pain intensity during short-term and long-term follow-up after TKA. Williams et al. [9] performed a meta-analysis that comprised 41 trials including 6255 patients, and the results revealed that the benefit of CBT on chronic pain in adults is minimal. Another meta-analysis by Ma et al. [13] included 6 high-quality randomized controlled trials, and the results also indicated that CBT did not improve postoperative pain compared to standard care. These findings are consistent with the results of our study. However, three studies included in this meta-analysis [11, 17, 18] reported that patients in the CBT intervention group had lower pain levels in the short term compared to those receiving standard care. This may be due to several reasons. Firstly, patients who undergo TKA are largely dependent on the surgical outcome, and individual differences between patients, varying degrees of soft tissue release and removal of osteophyte during surgery can lead to different levels of postoperative pain [26, 27]. In addition, the relatively limited sample size in this studies may have contributed to the differences in the efficacy of CBT; secondly, cognitive efficiency, as measured by processing speed and memory, tends to decline with age. Older adults are at an increased risk of developing cognitive impairments compared to younger adults [28–30], which may also affect the effectiveness of CBT interventions. Finally, upon further analysis of the studies included in this meta-analysis, we found that the three studies reporting positive results for CBT were all conducted in China [11, 17, 18], while the other two studies reporting no significant difference in pain reduction were carried out in Denmark and the USA [15, 16]. Differences in cultural and belief systems across regions may have also influenced the efficacy of CBT interventions. Research has shown that race, culture, and educational level are closely related to the response to pain stimuli [31–33]. Therefore, cultural and contextual factors may have played a role in the variability of the efficacy of CBT in reducing postoperative pain in TKA patients. We also noticed that the populations included in two studies [11, 18] were both diagnosed with kinesiophobia (TSK > 37), which may have influenced the effectiveness of CBT interventions. Differences in the characteristics of these populations may have also influenced the efficacy of CBT. The differences in population characteristics between these studies may also partly explain the heterogeneity observed in the results. However, it is important to recognize that false positives resulting from the small sample sizes of these studies may also be a contributing factor. By combining the data from each study, we were able to sufficiently expand the sample size, enhance the test efficacy of the studies, reduce Type II errors, and make the conclusions more reliable.

Currently, there is a lack of meta-analyses exploring the effects of CBT interventions on postoperative knee joint function in TKA patients. Out of the studies included in our analysis, only four studies reported on postoperative knee joint function, and the combined effect size indicates that CBT interventions do not lead to significant improvement in knee joint function after TKA surgery. Our findings align with those of Birch et al. [15]. In their study, which included 60 participants, no statistically significant difference in knee function scores were observed between the two groups at the 3-month and 1-year postoperative follow-up. However, two of the studies included in our analysis [11, 14] suggest that CBT interventions may improve early postoperative knee joint function. The heterogeneity test revealed significant statistical heterogeneity among the studies reporting short-term knee joint function outcomes in this meta-analysis. However, after sensitivity analysis, the combined effect size did not significantly change, indicating that the results were stable. Through our analysis of the included studies, we have identified potential reasons for the observed heterogeneity. Firstly, in the studies conducted by [12, 17], CBT interventions were carried out by doctors, nurses, and individual psychotherapists separately. In contrast, in the studies by [11, 14], CBT interventions were jointly conducted by physiotherapists and psychotherapists. The presence of physical therapists providing postoperative physical rehabilitation may have led to more effective implementation of CBT interventions [13], resulting in more favorable outcomes for knee joint function after TKA surgery. Secondly, the study of [12] included patients with an average age over 70 years, and cognitive efficiency tends to decline with age, which may affect the effectiveness of CBT interventions. These factors may have contributed to the differences in results observed between studies. Methodological heterogeneity among the studies may have also contributed to statistical heterogeneity. This suggests that the effectiveness of CBT interventions may not always be maintained under different influencing conditions.

Pain catastrophizing is a reliable psychological predictor of adverse outcomes after TKA surgery [34]. In the process of CBT intervention, patients are trained to recognize irrational catastrophic thoughts that may contribute to pain, and how to substitute them with rational thoughts, ultimately leading to improved postoperative pain. In this meta-analysis, three studies reported postoperative Pain Catastrophizing Scale (PCS) scores, with three reporting short-term results and two reporting long-term results. The combined effect size suggests that CBT intervention is not effective in reducing patients' pain catastrophizing levels compared to usual care. Our findings are consistent with a meta-analysis conducted by Ma et al. [13] which included six studies and found that CBT intervention did not significantly reduce pain catastrophizing levels in TKA patients within one year postoperatively. However, other studies have shown that CBT has clear therapeutic effects in reducing pain catastrophizing. A meta-analysis by Gibson et al. [35] found that CBT intervention can significantly reduce PCS scores. Through careful analysis, we noticed that most of the studies included in Gibson et al.'s research focused on chronic low back pain and chronic neuropathic pain patients, with only a few studies including TKA patients. Compared to other types of chronic pain patients, TKA patients experience more acute postoperative pain, and the effectiveness of CBT may not be significant in this population. Besides, among the three studies reporting short-term PCS scores, two studies [11, 17] reported that the CBT intervention group had lower PCS scores than the usual care group in the short term. However, we found that in another study, only highly pain catastrophizing patients (PCS ≥ 22) were included, and patients with higher PCS scores are more likely to experience more intense pain, which reduces the effectiveness of CBT intervention [36], Therefore, this may have led to different results among the studies, and this inevitably contributes to some degree of heterogeneity.

In this meta-analysis, three studies reported TSK scores in the short term after TKA, and the combined effect size results indicated that CBT intervention can effectively mitigate the fear of movement postoperatively. TKA patients often have fear of movement regarding the rehabilitation exercises for their knee joint, mainly due to concerns about potential injury or loss of function caused by exercising the knee joint during pain [37]. However, if early exercise is not carried out, it may lead to knee joint adhesions, muscle atrophy, and even deep venous thrombosis. Currently, CBT is widely used to treat patients with fear of movement. CBT changes patients' misconceptions from a cognitive perspective, conducts cognitive reconstruction, and intervenes and supports patients' behavior. Many studies have shown that CBT can help patients correct their negative cognition and behavior, increase their sense of self-control, and alleviate postoperative fear of movement [25]. However, in this study, we noticed that the advantage of CBT intervention in kinesiophobia may not have facilitated the recovery of knee joint function after TKA. We believe that the functional recovery of the knee joint after surgery requires long-term CBT intervention. However, the included studies mostly implemented CBT during hospitalization, and other psychological factors may also affect knee joint function [36].

The present study has several limitations: (1) Only 7 studies were included in the analysis, and the sample size is still limited, with most of the combined effect sizes based on only 3 studies; (2) Some patients may require longer-term intervention to correct long-standing and stubborn negative beliefs and behavioral patterns that have caused them prolonged suffering. Additionally, regular intervention over a long period may be necessary to maintain treatment stability for some patients; (3) The majority of the studies were conducted in the United States and China, with only one study conducted in Denmark. Hence, the generalizability of the results to TKA patients worldwide may be limited; (4) The outcome measures relied on subjective self-reported results, and the use of objective physiological measures may lead to different outcomes; (5) Finally, similar to other meta-analyses, some unpublished studies may have been missed. Future research should further analyze and validate these limitations.

## Conclusions

Our study suggests that although CBT can alleviate the degree of kinesiophobia in the early postoperative period of TKA patients, but did not significantly relieve knee pain or improve knee function. Further research, including multi-center, large-sample RCT studies, is needed to analyze the long-term efficacy of CBT and to consider the effects of different patient characteristics on CBT intervention.

### Supplementary Information

**Supplementary Material 1.**

**Supplementary Material 2.**

**Supplementary Material 3.**

## Data Availability

Upon reasonable request, the data from this study can be obtained by contacting the corresponding author.
