# Supplementary material for: Effect of cognitive behavioral therapy on pain, knee function, and psychological status in patients after primary total knee arthroplasty: a systematic review and meta-analysis
Source: BMC Musculoskelet Disord. 2024 Apr 11;25:280. doi: 10.1186/s12891-024-07413-1 (PMC11007994; doi:10.1186/s12891-024-07413-1)

PubMed search strategy

Search:((("Cognitive Behavioral Therapy"[Mesh]) OR (((((((((((((((((((((((((((((Behavioral Therapies, Cognitive[Title/Abstract]) OR (Behavioral Therapy, Cognitive[Title/Abstract])) OR (Cognitive Behavioral Therapies[Title/Abstract])) OR (Therapies, Cognitive Behavioral[Title/Abstract])) OR (Therapy, Cognitive Behavioral[Title/Abstract])) OR (Behavior Therapy, Cognitive[Title/Abstract])) OR (Cognitive Behavior Therapy[Title/Abstract])) OR (Cognitive Behaviour Therapy[Title/Abstract])) OR (Behaviour Therapies, Cognitive[Title/Abstract])) OR (Behaviour Therapy, Cognitive[Title/Abstract])) OR (Cognitive Behaviour Therapies[Title/Abstract])) OR (Therapies, Cognitive Behaviour[Title/Abstract])) OR (Therapy, Cognitive Behaviour[Title/Abstract])) OR (Cognitive Therapy[Title/Abstract])) OR (Therapy, Cognitive Behavior[Title/Abstract])) OR (Behavior Therapies, Cognitive[Title/Abstract])) OR (Cognitive Behavior Therapies[Title/Abstract])) OR (Therapies, Cognitive Behavior[Title/Abstract])) OR (Cognitive Psychotherapy[Title/Abstract])) OR (Cognitive Psychotherapies[Title/Abstract])) OR (Psychotherapies, Cognitive[Title/Abstract])) OR (Psychotherapy, Cognitive[Title/Abstract])) OR (Therapy, Cognitive[Title/Abstract])) OR (Cognitive Therapies[Title/Abstract])) OR (Therapies, Cognitive[Title/Abstract])) OR (Therapy, Cognition[Title/Abstract])) OR (Cognition Therapy[Title/Abstract])) OR (Cognition Therapies[Title/Abstract])) OR (Therapies, Cognition[Title/Abstract]))) AND (("Arthroplasty, Replacement, Knee"[Mesh]) OR (((((((((((((((Arthroplasties, Replacement, Knee[Title/Abstract]) OR (Arthroplasty, Knee Replacement[Title/Abstract])) OR (Knee Replacement Arthroplasties[Title/Abstract])) OR (Knee Replacement Arthroplasty[Title/Abstract])) OR (Replacement Arthroplasties, Knee[Title/Abstract])) OR (Knee Arthroplasty, Total[Title/Abstract])) OR (Arthroplasty, Total Knee[Title/Abstract])) OR (Total Knee Arthroplasty[Title/Abstract])) OR (Replacement, Total Knee[Title/Abstract])) OR (Total Knee Replacement[Title/Abstract])) OR (Knee Replacement, Total[Title/Abstract])) OR (Knee Arthroplasty[Title/Abstract])) OR (Arthroplasty, Knee[Title/Abstract])) OR (Arthroplasties, Knee Replacement[Title/Abstract])) OR (Replacement Arthroplasty, Knee[Title/Abstract])))) AND (((randomized controlled trial[Title/Abstract]) OR (randomized[Title/Abstract])) OR (placebo[Title/Abstract]))

Embase search strategy

| No. | Query | Results | Date |
| --- | --- | --- | --- |
| #8 | #3 AND #6 AND #7 | 11 | 20-Oct-22 |
| #7 | 'randomized controlled trial':ab,ti OR 'randomized':ab,ti OR 'placebo':ab,ti | 1075067 | 20-Oct-22 |
| #6 | #4 OR #5 | 47492 | 20-Oct-22 |
| #5 | 'arthroplasties, replacement, knee':ab,ti OR 'arthroplasty, knee replacement':ab,ti OR 'knee replacement arthroplasties':ab,ti OR 'knee replacement arthroplasty':ab,ti OR 'knee arthroplasty, total':ab,ti OR 'replacement arthroplasties, knee':ab,ti OR 'arthroplasty, total knee':ab,ti OR 'total knee arthroplasty':ab,ti OR 'replacement, total knee':ab,ti OR 'total knee replacement':ab,ti OR 'knee replacement, total':ab,ti OR 'knee arthroplasty':ab,ti OR 'arthroplasty, knee':ab,ti OR 'arthroplasties, knee replacement':ab,ti OR 'replacement arthroplasty, knee':ab,ti | 39782 | 20-Oct-22 |
| #4 | 'total knee arthroplasty'/exp | 33773 | 20-Oct-22 |
| #3 | #1 OR #2 | 33067 | 20-Oct-22 |
| #2 | 'behavioral therapies, cognitive':ab,ti OR 'behavioral therapy, cognitive':ab,ti OR 'cognitive behavioral therapies':ab,ti OR 'therapies, cognitive behavioral':ab,ti OR 'therapy, cognitive behavioral':ab,ti OR 'behavior therapy, cognitive':ab,ti OR 'cognitive behavior therapy':ab,ti OR 'cognitive behaviour therapy':ab,ti OR 'behaviour therapies, cognitive':ab,ti OR 'behaviour therapy, cognitive':ab,ti OR 'cognitive behaviour therapies':ab,ti OR 'therapies, cognitive behaviour':ab,ti OR 'therapy, cognitive behaviour':ab,ti OR 'cognitive therapy':ab,ti OR 'therapy, cognitive behavior':ab,ti OR 'behavior therapies, cognitive':ab,ti OR 'cognitive behavior therapies':ab,ti OR 'therapies, cognitive behavior':ab,ti OR 'cognitive psychotherapy':ab,ti OR 'cognitive psychotherapies':ab,ti OR 'psychotherapies, cognitive':ab,ti OR 'psychotherapy, cognitive':ab,ti OR 'therapy, cognitive':ab,ti OR 'cognitive therapies':ab,ti OR 'therapies, cognitive':ab,ti OR 'therapy, cognition':ab,ti OR 'cognition therapy':ab,ti OR 'cognition therapies':ab,ti OR 'therapies, cognition':ab,ti | 12766 | 20-Oct-22 |
| #1 | 'cognitive behavioral therapy'/exp | 22854 | 20-Oct-22 |

Cochrane search strategy


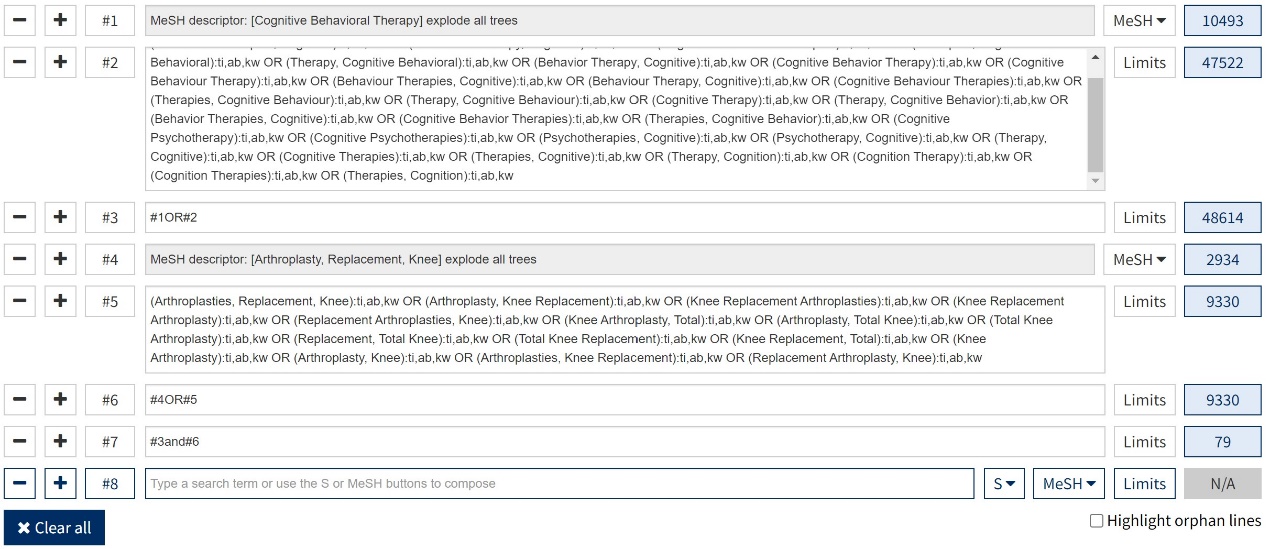

Supplement: Supplementary file 2 — Supplementary Material 2. [file 12891_2024_7413_MOESM2_ESM.docx]
