# Supplementary material for: Effect of cognitive behavioral therapy on pain, knee function, and psychological status in patients after primary total knee arthroplasty: a systematic review and meta-analysis
Source: BMC Musculoskelet Disord. 2024 Apr 11;25:280. doi: 10.1186/s12891-024-07413-1 (PMC11007994; doi:10.1186/s12891-024-07413-1)
Supplement: Supplementary file 3 — Supplementary Material 3. [file 12891_2024_7413_MOESM3_ESM.docx]

| Indicators of outcome for studies included in this meta-analysis | TSK | EG CG | NM | NM | NM | 34.91±6.61 43.35±5.67  NM | NM | 38.90±5.07 44.18±5.83  NM | 30.94±2.65 36.74±3.29  NM | Note: EG: Experimental group; CG: Control Group; NM: Not Mentioned; PCS: Pain Catastrophizing Scale; TSK: Tampa Scale of Kinesiophobia |
| --- | --- | --- | --- | --- | --- | --- | --- | --- | --- | --- |
|  | PCS | EG CG | 13.0±10.2 11.0±10.2  11.0±11.0 9.0±11.5 | NM | 14.2±5.9 17.3±6.2  10.4±1.9 11.1±3.6 | NM | NM | 31.82±3.70 33.52±4.34  NM | NM  NM |  |
|  | Knee function | EG CG | NM | NM | 73.6±6.7 74.7±6.0  88.9±6.8 87.8±6.5 | 69.57±12.49 63.83±7.64  NM | 73.9±7.7 74.8±8.0  89.3±8.6 88.9±8.2 | 62.58±6.60 56.36±7.92  NM | NM |  |
|  | Pain during activity | EG CG | 22.0±20.0 15.0±18.7  12.0±16.2 9.0±15.6 | 2.3±2.2 2.3±2.2  NM | 1.5±0.7 1.9±1.0  0.8±0.6 0.9±0.6 | NM | NM | NM | 2.08±0.63 2.34±0.66 |  |
|  | Pain during rest | EG CG | 11.0±12.5 7.0±11.5  7.0±13.7 6.0±14.3 | 1.3±2.2 1.0±1.5  NM | NM | NM | NM | 6.23±0.65 6.52±0.77  NM | NM |  |
|  | follow-up  periods | | Short term  Long term | Short term  Long term | Short term  Long term | Short term  Long term | Short term  Long term | Short term  Long term | Short term  Long term |  |
|  | Study  (Author, year) | | Birch et al. (2020) | Buvanendran et al. (2017) | Sun et al. (2020) | Cai et al. (2017) | Chen et al. (2021) | Cai et al. (2018) | Qian et al. (2023) |  |
|  | No. | | 1 | 2 | 3 | 4 | 5 | 6 | 7 |  |
